# ComTarget: Small-Molecule Target Prediction with Combinatorial Modeling

**DOI:** 10.3390/ph19050715

**Published:** 2026-04-30

**Authors:** Yuzhu Li, Qingyi Shi, Xingjie Lu, Daiju Yang, Dilixiati Yeerken, Huizi Jin, Qingyan Sun

**Affiliations:** 1 School of Pharmaceutical Sciences, Shanghai Jiao Tong University, Shanghai 200240, China; 2 National Key Laboratory of Lead Druggability Research, Shanghai Institute of Pharmaceutical Industry, China State Institute of Pharmaceutical Industry, China State Institute of Pharmaceutical Industry Co., Ltd., Shanghai 201203, China

**Keywords:** ComTarget, 3D molecular similarity, reverse docking, target prediction

## Abstract

**Background**: Identifying potential targets for bioactive compounds is crucial for elucidating the mechanisms of action and drug development. **Methods**: This study presents ComTarget, a computational tool that integrates 3D molecular shape similarity analysis (based on combined 3D descriptors, C3DD) with reverse docking to predict protein targets for small molecules. ComTarget screens against a library of 4429 unique protein targets derived from 26,272 PDB complexes. **Results:** Validation on benchmark datasets (DEKOIS 2.0 and DUDE-Z) demonstrated that the C3DD molecular similarity calculation method effectively enriches active ligands by capturing critical 3D shape information not evident from chemical topology alone. It outperformed conventional 2D fingerprint methods and offered a favorable balance between shape sensitivity and computational efficiency, serving as a rapid pre-screening filter within the integrated workflow. For FDA-approved drugs (e.g., Imatinib, Aspirin) and natural products (e.g., Berberine). ComTarget identified targets consistent with reported therapeutic targets or putative off-targets in the literature, while also revealing potential targets aligned with the compounds’ pharmacological mechanisms. **Conclusions**: As a local program, ComTarget offers flexibility in computational resources customization and is freely available for polypharmacology studies, drug repurposing, and adverse reaction prediction.

## 1. Introduction

The identification of potential targets for bioactive compounds is fundamental to drug design and development. However, many compounds fail in clinical trials or are withdrawn from the market due to toxic side effects observed during clinical testing [1]. Modern drug discovery heavily focuses on developing drugs that act on specific targets with high potency and selectivity. However, it is increasingly recognized that these concepts may be oversimplified, which makes it difficult to explain the mechanisms of certain drugs or design therapies for complex multifactorial diseases. With advancing insights into biological systems and disease associations, the concept of polypharmacology has gained traction. Designing single drug molecules that can simultaneously and specifically act on multiple targets is becoming an important direction in drug development [2,3,4]. Nevertheless, the development of multi-target drugs presents considerable challenges [2], particularly because experimentally identifying the actual targets from a vast pool is costly. Experimental techniques for studying drug-target interactions include Affinity Chromatography, two-dimensional gel electrophoresis (2DE), Drug Affinity Responsive Target Stability (DARTS), Target identification by Chromatographic Co-Elution (TICC), protein microarrays, and others [5,6]. However, these experimental approaches are relatively expensive in terms of resources and time, making computer-aided target prediction a crucial alternative to experimental target identification.

Computer-aided target prediction, also referred to as target fishing (or computer reverse screening), primarily aims to identify the most probable targets for a query molecule. This approach can predict potential drug-target interactions and the mechanism of action of bioactive molecules, forecast possible adverse drug reactions, evaluate polypharmacology, and hold significant potential in drug repurposing. It can be categorized into three types: ligand-based methods, receptor-based methods, and the combined ligand- and receptor-based strategies.

A variety of computational tools have been developed for ligand-based target prediction. Tools relying on 2D ligand similarity include MolTarPred [7], TargetHunter [8], TarPred [9], and MuSSeL [10]. For 3D ligand similarity, CSNAP3D is a representative tool, while SwissTargetPrediction integrates both 2D and 3D molecular similarity approaches [11,12]. In the realm of machine learning, tools encompass Multi-target QSAR models [13], random forest-based QSAR models [14], and deep learning frameworks such as DeepDTIs and STarFish [15,16]. Keiser et al. developed the chemical similarity ensemble approach (SEA), a method that quantitatively groups and relates proteins based on the chemical similarity of their ligands [17]. Subsequently, the SEA and similar methodologies have been effectively utilized in identifying new targets for existing drugs and natural products [17,18,19,20], predicting side effects [21].

In receptor-based target prediction, computational tools have been developed that primarily rely on two main strategies: reverse docking and pharmacophore-based target fishing. Tools adopting the reverse docking strategy include idTarget [22], TarFisDock [23], DPDR-CPI [24], INVDOCK [25], and ACID [26]. The field also encompasses specialized tools for specific protein families, such as DIA-DB [27], a web server for antidiabetic drug targets, and GUT-DOCK for G protein-coupled receptors [28]. However, reverse docking faces several challenges, including the requirement to construct appropriate target datasets, high computational costs associated with modeling receptor flexibility, and limited accuracy in binding free energy predictions for target ranking. As a complementary strategy, pharmacophore-based methods utilize key molecular interaction features for target identification. Commonly used tools include PharmMapper [29], Drug ReposER [30], LigAdvisor, and PLIP [31,32]. Compared with reverse docking, these methods generally offer faster screening speeds and are less dependent on high-quality protein structures, though they may provide less detailed binding modes information.

Each computational strategy, whether ligand-based or receptor-based, has its own advantages and limitations. Ligand-based methods are constrained by the limited coverage of known chemical information and face challenges in reliably ranking predictions and setting thresholds. In contrast, receptor-based methods face constraints due to limited protein structure data and inaccuracies in docking scores. To tackle these challenges, integrating both strategies holds promise, as it combines ligand similarity with structural features of target receptors to yield a more comprehensive understanding of drug-target interactions. Many existing tools are offered primarily as web services, which can restrict their adaptability to meet diverse and tailored research requirements.

To address these challenges, we developed ComTarget, a localized tool that integrates 3D molecular surface shape similarity comparison with reverse docking. This combined approach leverages the complementary strengths of these two approaches, offering an efficient solution for target prediction. ComTarget is freely available for download from https://github.com/CalVSP/ComTarget.git (accessed on 25 April 2026).

**Figure 1:**
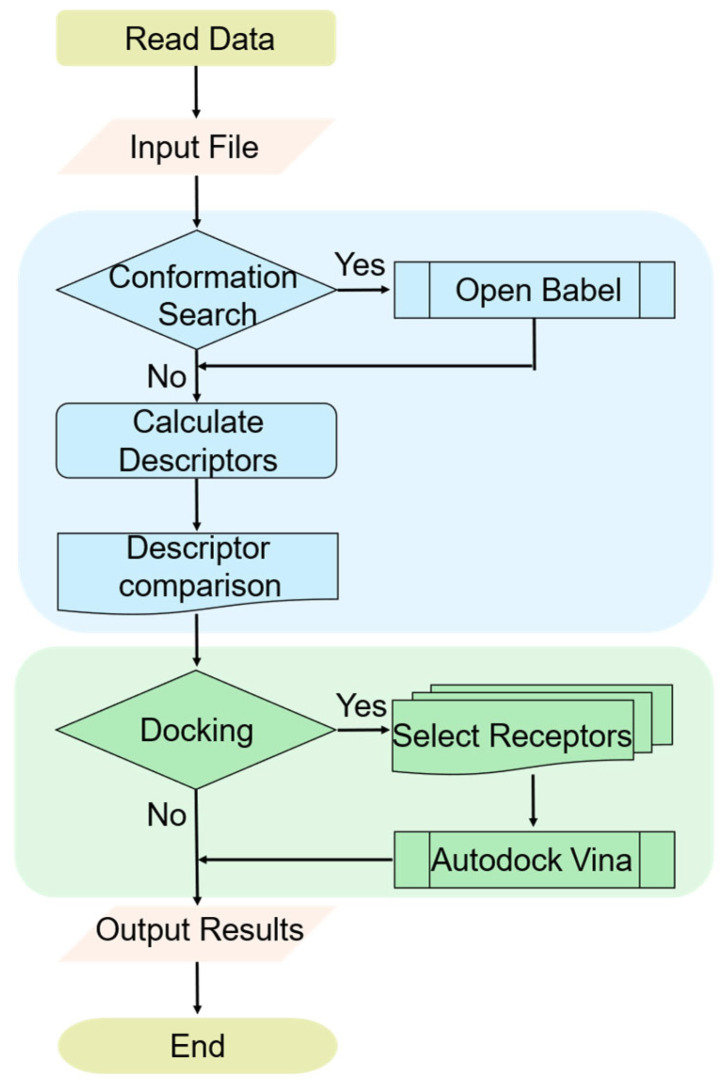
Flow chart presenting the ComTarget calculation algorithm. Arrows indicate the program workflow. Colors represent different functional modules: light blue for molecular similarity calculation, light green for reverse docking, PaleGoldenrod for start/end, and MistyRose for file reading/output.

The ComTarget workflow integrates two sequential stages: a rapid ligand-based pre-screening and a precise structure-based reverse docking (Figure 1). In the first stage, the query molecule is processed to extract three-dimensional shape descriptors (C3DD), which are then compared against a pre-computed library of ligand conformations derived from the Protein Data Bank (PDB). This similarity search efficiently narrows the target space to a manageable list of high-probability candidates. Subsequently, the second stage performs reverse docking of the query molecule into the binding pockets of the shortlisted targets using AutoDock Vina 1.1.2 (11 May 2011). Finally, a ranked list of candidate targets is generated.

Our benchmark evaluations and case studies demonstrate that ComTarget effectively retrieves known therapeutic targets and identifies potential off-target interactions by combining efficient 3D shape-based pre-screening with physics-based docking, highlighting its utility as a scalable, open-source platform for drug discovery to explore complex mechanisms of action and advance multi-target drug development.

## 2. Results

### 2.1. Evaluation of C3DD in Ligand Similarity Search

To evaluate the C3DD molecular shape descriptors, we performed benchmarking on two rigorously constructed datasets, DEKOIS 2.0 and DUDE-Z [33,34], which minimizes scaffold bias between actives and decoys. We compared C3DD with 2D finger-prints (ECFP4, MACCS) and 3D shape-based methods (shapescreen (version 1.2.3), ROSHAMBO (version 0.0.1)) [7,35,36]. Performance was assessed using AUC and enrichment factor at 5% (EF5%).

#### 2.1.1. Performance on the DEKOIS 2.0 Benchmark

**Figure 2:**
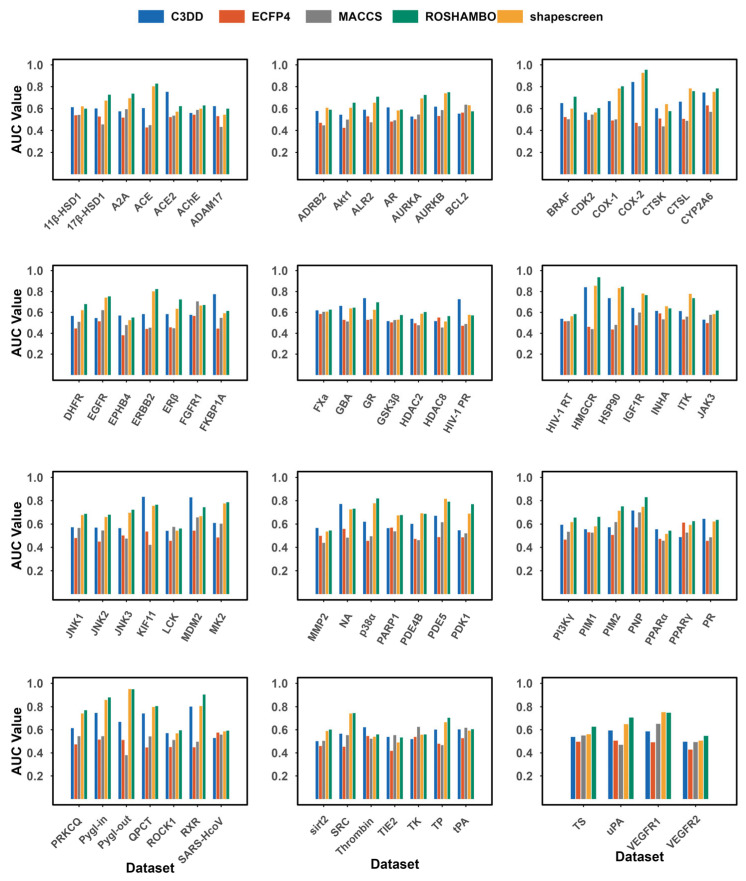
Areas under the ROC curves comparison of the C3DD method with ECFP4, MACCS, shapescreen (version 1.2.3), and ROSHAMBO (version 0.0.1) tested on the DEKOIS_2.0 dataset. ROSHAMBO software employs the ComboTanimoto score evaluation method.

To quantify the overall discriminatory power of C3DD as a classifier for identifying active molecules, we calculated the ROC curve AUC for each target in the DEKOIS benchmark (Figure 2). C3DD achieved an average AUC of 0.617, which is above the random classification baseline of 0.5. C3DD’s mean performance was between the 2D fingerprints (ECFP4: 0.500, MACCS: 0.527) and 3D shape methods (shapescreen: 0.664, ROSHAMBO: 0.690). This result establishes C3DD as a practically useful balance between computational efficiency and 3D-informative power.

C3DD demonstrated advantages on specific targets where 3D shape complementarity is likely crucial, such as ACE2 (0.752), Aurora B (0.740), and KIF11 (0.833), often outperforming 2D fingerprints by a significant margin. This highlights its ability to identify similarities that are not evident from chemical topology alone.

Our comparative analysis demonstrates that C3DD provides a valuable performance profile among contemporary similarity methods. While its mean AUC is lower than that of the sophisticated 3D alignment tool ROSHAMBO (0.690), it consistently outperforms conventional 2D fingerprints (ECFP4: 0.500; MACCS: 0.527). This indicates that C3DD successfully captures critical 3D shape information that is inherently missed by substructure-based approaches, thereby overcoming a key limitation of 2D methods by identifying actives with dissimilar scaffolds but similar bioactive conformations.

**Figure 3:**
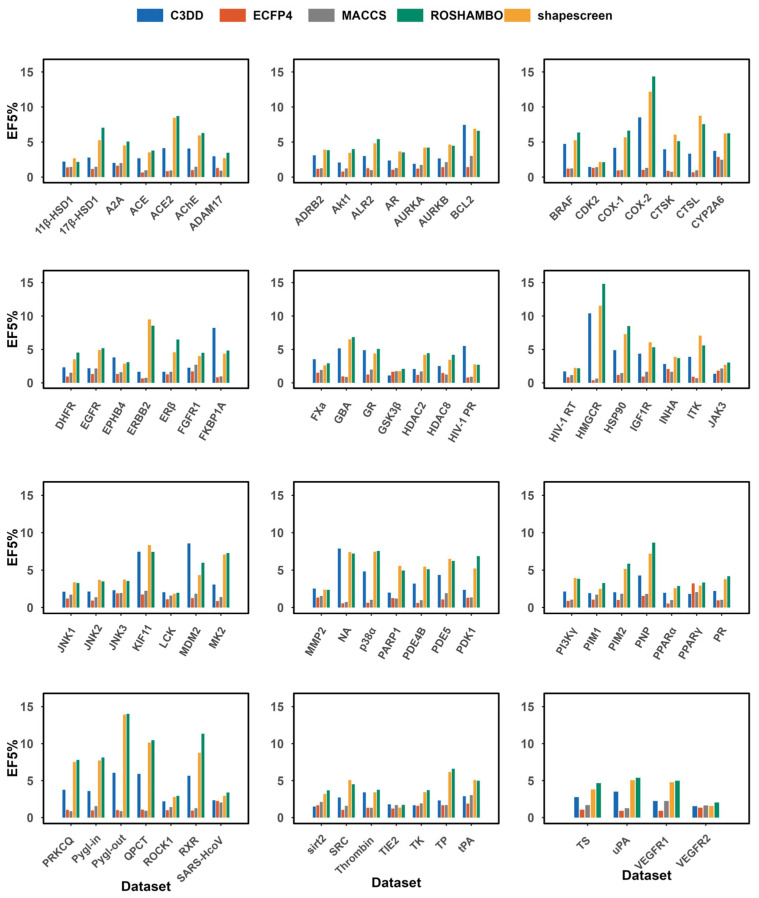
Enrichment Factor at 5% comparison of the C3DD method with ECFP4, MACCS, shapescreen (version 1.2.3), and ROSHAMBO (version 0.0.1) tested on the DEKOIS_2.0 dataset. ROSHAMBO software employs the ComboTanimoto score evaluation method.

Beyond overall ranking accuracy, the practical utility of a virtual screening method heavily relies on its ability to prioritize active compounds at the very top of the ranked list. We therefore evaluated the early enrichment performance using the enrichment factor at 5% (EF5%). As shown in Figure 3 and summarized by the average values (C3DD: 3.42; ECFP4: 1.21; MACCS: 1.50; shapescreen: 5.05; ROSHAMBO: 5.37), C3DD again demonstrated an advantage over conventional 2D fingerprints, with an average EF5% nearly threefold higher than that of ECFP4. This substantial gap underscores the critical role of 3D shape information in achieving meaningful early enrichment, a task where topology-based methods often struggle.

C3DD achieved early enrichment on several targets, such as HMGCR (EF5% = 10.42), ACE2 (4.13), and FKBP1A (8.23), where it outperformed all 2D methods and even rivaled the performance of the ROSHAMBO method on FKBP1A. These results indicate that for specific protein targets where ligand binding is highly shape-sensitive, the C3DD descriptors can effectively concentrate true actives within the first few percent of the screening library, a key requirement for lead identification campaigns. While the 3D shape alignment methods (shapescreen and ROSHAMBO) yielded higher average EF5% values, C3DD provides a favorable trade-off, delivering competent early enrichment (substantially better than 2D methods) alongside the computational efficiency and simplicity of a descriptor-based approach.

#### 2.1.2. Performance on the DUDE-Z Benchmark

**Figure 4:**
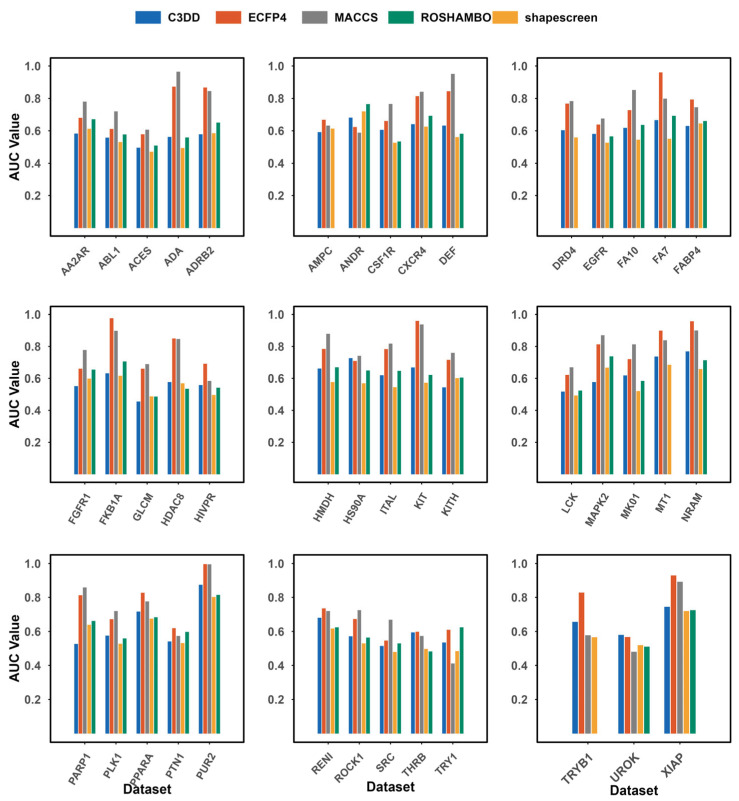
Areas under the ROC curves comparison of the C3DD method with ECFP4, MACCS, shapescreen (version 1.2.3), and ROSHAMBO (version 0.0.1) tested on the DUDE-Z dataset. ROSHAMBO software employs the ComboTanimoto score evaluation method. Due to calculation failures, data for the ROSHAMBO targets (AMPC, DRD4, MT1, and TRYB1) are missing.

To ensure broad validation, we evaluated the methods on the distinct DUDE-Z benchmark. As summarized in Figure 4, C3DD achieved an average AUC of 0.613 on the DUDE-Z dataset. Its overall mean performance is below that of the 2D fingerprints (ECFP4: 0.752; MACCS: 0.757). However, it significantly outperformed the rapid shape-screening method, shapescreen (0.577), and demonstrated performance close to the advanced shape-pharmacophore hybrid method ROSHAMBO (0.619).

**Figure 5:**
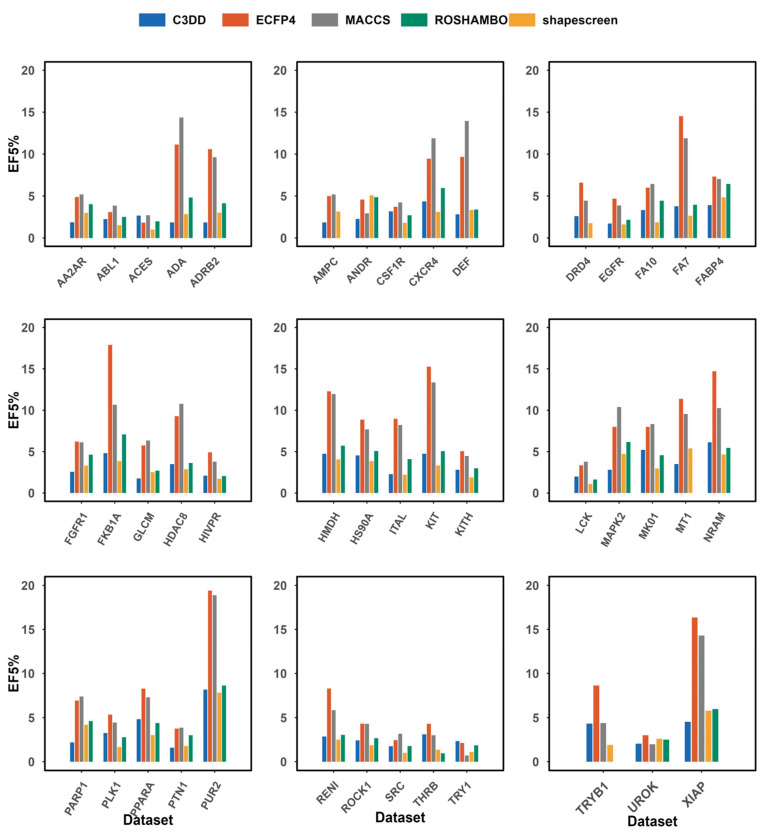
Enrichment Factor at 5% comparison of the C3DD method with ECFP4, MACCS, shapescreen (version 1.2.3), and ROSHAMBO (version 0.0.1) tested on the DUDE-Z dataset. ROSHAMBO software employs the ComboTanimoto score evaluation method.

The early enrichment results on DUDE-Z (Figure 5) provide further insight. The average EF5% of C3DD (3.19) was lower than that of the 2D fingerprints (ECFP4: 7.82; MACCS: 7.28), reflecting the challenge for shape-based methods on this set, where actives may share substructural motifs. However, within the category of 3D methods, C3DD outperformed shapescreen (2.92) and approached the performance of ROSHAMBO (3.96). Furthermore, C3DD achieved strong early enrichment on specific targets such as PUR2 (EF5% = 8.18), NRAM (6.14), and MK01 (5.22), demonstrating its efficacy when shape complementarity is key.

The primary value of C3DD within the ComTarget workflow lies in its optimal balance between shape sensitivity and computational efficiency. Unlike alignment-intensive methods like ROSHAMBO, C3DD’s moment-invariant descriptors require no molecular superposition, granting it a significant speed advantage for screening large libraries. Therefore, while it may not match the peak discriminative power of the most computationally expensive 3D tools in every case, its combination of reasonable accuracy, 3D awareness, and high throughput makes it an exceptionally effective pre-screening filter. Its role is to efficiently reduce the vast target- or ligand-space to a manageable set of high-probability candidates, which are then prioritized by the more precise, physics-based reverse docking stage.

### 2.2. Reverse Docking Section

We constructed a library of protein-ligand complexes encompassing 4429 unique protein targets (derived from 26,272 PDB entries). To establish a baseline and illustrate the intrinsic variation in docking scores across different systems, we redocked the original ligands into their native receptors using AutoDock Vina. The resulting docking scores for all 26,272 complexes spanned a broad range from −42.2 to −1.5 kcal/mol, confirming that comparisons of raw scores are unreliable for ranking ligands across diverse targets. Therefore, in all subsequent analyses, the normalized differential score (*DetScore*) as defined in Section 4.7 was used to prioritize potential targets for query compounds. This approach corrects for target-specific scoring biases and prioritizes targets where the query molecule exhibits improved binding affinity relative to the native co-crystallized ligand.

### 2.3. Effectiveness Validation of Comtarget

This validation was designed to assess the ability of the integrated workflow to correctly identify known targets for diverse query ligands across a broad range of protein families by combining 3D molecular shape similarity pre-screening (C3DD) with reverse docking.

We selected 23 therapeutically relevant protein targets spanning major drug target classes, including kinases (e.g., BRAF, EGFR, SRC), enzymes (e.g., COX-1, COX-2, DHFR, thrombin), and nuclear receptors (e.g., ERβ). For each target, three established active ligands were used as query molecules, resulting in a total of 69 queries. The ranked list of targets was analyzed for its ability to recall the known true target of each query ligand. Performance was quantified using the AUC and early enrichment factors (EF1% and EF5%).

**Figure 6:**
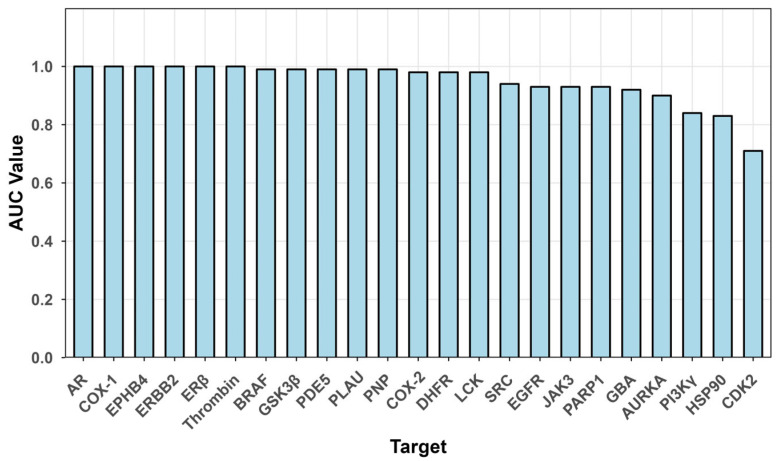
Areas under the ROC curves comparison of the Comtarget on the 23 targets.

The ComTarget workflow correctly retrieved the known true target for all 69 query ligands (100% recall), meaning the correct target was ranked within the top 200 in the integrated workflow’s output. The prediction accuracy, measured by AUC, was consistently high across the 23 targets (Figure 6). The mean AUC was 0.950 ± 0.071 (median = 0.98), with 13 out of 23 targets achieving an AUC ≥ 0.98 and 7 targets reaching an AUC of 1.00.

**Figure 7:**
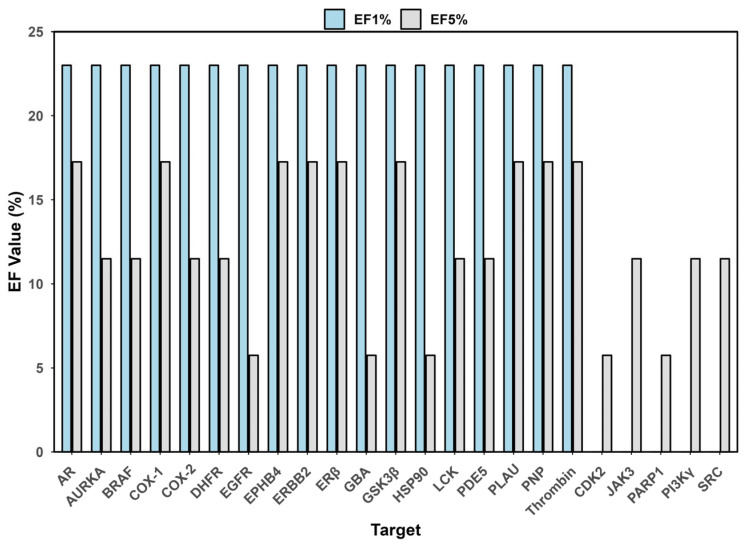
Enrichment Factor at 1% and 5% of the Comtarget on the 23 targets.

Early enrichment capability, which is critical for practical virtual screening, was particularly strong for ComTarget. The average enrichment factor (Figure 7) at the top 1% and 5% of the screened list were EF1% = 18.0 and EF5% = 12.5, respectively. This represents that ComTarget enriches true positive targets by more than 12-fold in the top 5% of predictions compared to random selection, highlighting the method’s efficiency in rapidly focusing on the most promising targets.

This expanded benchmark, encompassing 23 targets and 69 query ligands, confirms ComTarget as an effective and reliable tool for computational target prediction. The integrated ComTarget workflow, which ensures high recall and precision, is well-suited for applications in polypharmacology mapping, drug repurposing, and off-target identification.

### 2.4. Test for Descriptor Calculation Runtime

The workflow of ComTarget consists of three main steps: calculation of 3D similarity descriptors for the query molecule, similarity search against the 3D molecular descriptor library, and reverse docking.

**Figure 8:**
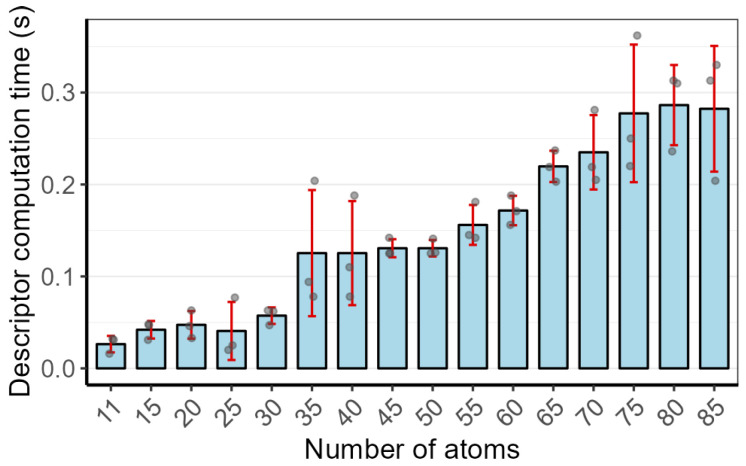
Calculate the descriptor time for molecules with different atomic numbers. Circles: individual data points; red lines: error bars.

In the descriptor calculation step, the computational time increases slightly with the number of atoms in the molecule (Figure 8). For molecules with fewer than 50 atoms, the calculation takes under 0.15 s, and for those with fewer than 100 atoms, it remains under 0.4 s. The similarity search against the 3D molecular descriptor library requires approximately 0.16 s, which is practically negligible. In contrast, the reverse docking step for each submitted molecule is the most time-consuming. Its duration depends critically on factors such as molecular size, the number of rotatable bonds, and the size of the docking box.

### 2.5. Test Cases

**Figure 9:**
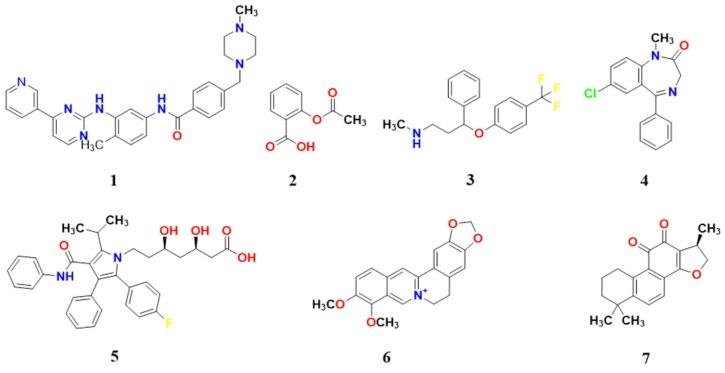
The structures of the tested bioactive molecules. Atom colors: N blue, O red, F yellow, Cl green.

We selected five common and important representative drugs: Imatinib (Figure 9, compound **1**), Aspirin (Figure 9, compound **2**), Fluoxetine (Figure 9, compound **3**), Diazepam (Figure 9, compound **4**), and Atorvastatin (Figure 9, compound **5**). These drugs cover multiple target categories (e.g., receptors, ion channels, enzymes) and therapeutic areas, enabling evaluation of the generalization ability of the ComTarget prediction method. Additionally, we tested two representative natural products: berberine (Figure 9, compound **6**) and cryptotanshinone (Figure 9, compound **7**).

#### 2.5.1. Imatinib

Imatinib (Figure 9, compound **1**) is a multi-target tyrosine kinase inhibitor. ComTarget’s predictions for imatinib encompassed targets across the full spectrum of binding affinities (Table 1), providing a case study for evaluating predictions against known pharmacology.

**Figure 10:**
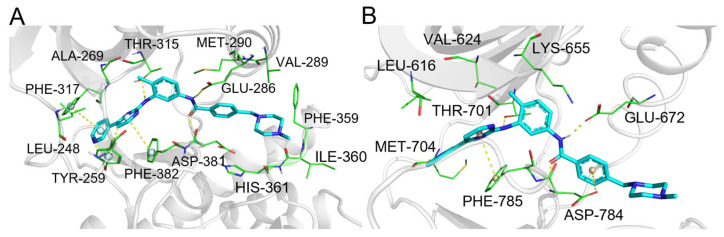
(**A**) Binding mode of imatinib and tyrosine protein kinase ABL1. (**B**) Binding mode of imatinib and epithelial discoidin domain-containing receptor 1. Color coding: protein: gray cartoon; key residues: green lines; imatinib: cyan sticks; π-π interactions: palecyan/wheat spheres; intermolecular interactions: yellow dashed lines.

The tool effectively prioritized several of its high- to moderate-affinity primary and secondary therapeutic targets (Categories I and II). These include the well-established targets ABL1 (ranked 1st), PDGFRA (10th), and KIT (58th), as well as other clinically relevant kinases such as SRC, LCK, EGFR, and BRAF, all ranking within the top 30. The predicted binding modes for ABL1 and DDR1 (Figure 10A,B) recapitulate key interactions, demonstrating molecular-level predictions.

ComTarget identified targets with weaker reported affinities (Category III). For instance, the prediction for MAPK14 is supported by biochemical data showing inhibition only at high micromolar concentrations (IC_50_ > 10 µM) [42]. While such interactions are unlikely to be pharmacologically relevant at therapeutic doses, their identification highlights the method’s sensitivity in detecting low-affinity binding. Notably, carbonic anhydrase 2 (CA2) was also predicted (Category I, based on a reported Kd of 30.2 nM). Overall, ComTarget recapitulated imatinib’s complex polypharmacology, highlighting that its predictions must be interpreted in conjunction with experimental affinity data.

#### 2.5.2. Aspirin

Aspirin (Figure 9, compound **2**) is a nonsteroidal anti-inflammatory drug (NSAID) whose primary therapeutic targets are cyclooxygenase-1 and -2 (COX-1/2). It is noteworthy that COX-1/2 were not among the top-ranked predictions, which is likely due to the limited availability of aspirin-bound crystal structures in the PDB data. Nevertheless, ComTarget identified several secondary targets (Table 2), which we have critically evaluated based on the strength of supporting evidence.

**Figure 11:**
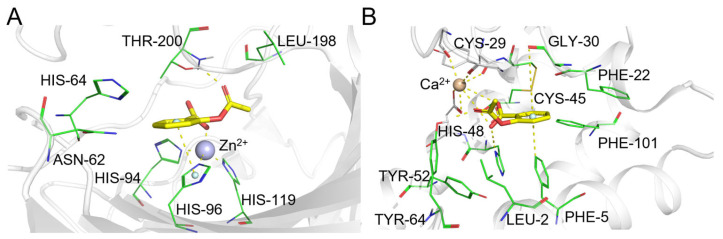
(**A**) Binding mode of aspirin and carbonic anhydrase 2. (**B**) The binding mode of aspirin and PLA2. Color coding: protein: gray cartoon; key residues: green lines; aspirin: yellow sticks; π-π interactions and p-π interactions: palecyan spheres; zinc ions: lightblue spheres; calcium ions: wheat spheres; intermolecular interactions: yellow dashed lines.

A notable prediction is the interaction with phospholipase A2 (PLA2) (ranked 60th, Category II). This is supported by a high-resolution co-crystal structure (1.9 Å) demonstrating direct binding of aspirin in the enzyme’s hydrophobic channel, with a reported dissociation constant (Kd) of 6.4 µM [46]. The predicted binding mode (Figure 11B) is consistent with this structural data, validating ComTarget’s ability to identify meaningful, medium-affinity off-target interactions.

Other predictions, such as those for CA2 and acetylcholinesterase, fall into Category III. For CA2, predicted binding mode (Figure 11A), literature indicates that inhibition is associated with aspirin’s metabolite, salicylic acid, at relatively high concentrations (mM range) [44]. Regarding AChE, a recent preclinical study reported inhibition only at very high doses of aspirin (100–300 mg/kg) [45]. This case demonstrates ComTarget’s ability to recapitulate validated off-targets while generating hypotheses about weaker interactions, underscoring the need to couple predictions with rigorous evidence assessment.

#### 2.5.3. Fluoxetine

**Figure 12:**
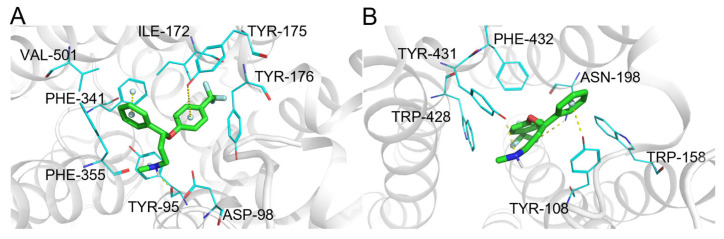
(**A**) Binding mode of fluoxetine and sodium-dependent serotonin transporter. (**B**) Binding mode of fluoxetine and histamine H1 receptor. Color coding: protein: gray cartoon; key residues: cyan lines; fluoxetine: green sticks; π-π interactions and p-π interactions: palecyan spheres; intermolecular interactions: yellow dashed lines.

Fluoxetine (Figure 9, compound **3**) is a selective serotonin reuptake inhibitor (SSRI). ComTarget successfully prioritized its primary therapeutic target, the serotonin transporter (SERT, Category I), which was ranked highly by both sorting methods (Table 3). The predicted binding mode of fluoxetine within SERT is shown in Figure 12A, illustrating key interactions consistent with its inhibitory function.

Beyond SERT, ComTarget also identified several targets with validated functional relevance (Category II). These include the histamine H1 receptor (HRH1), a prediction consistent with fluoxetine’s known sedative side effects. The complementary binding pose predicted for this interaction is shown in Figure 12B. Additionally, CA2 was identified, which fluoxetine potently activates (rather than inhibits) at clinically relevant concentrations (~1 µM) [51]. Notably, the prediction for the engineered bacterial leucine transporter (LeuBAT) underscores ComTarget’s ability to recognize the conserved binding architecture shared by the SLC6 neurotransmitter transporter family, as revealed by high-resolution co-crystal structures [50]. Additionally, ComTarget predicted binding to albumin (Category III), a nonspecific carrier protein, illustrating its ability to profile targets across the full spectrum of evidence.

#### 2.5.4. Diazepam

**Figure 13:**
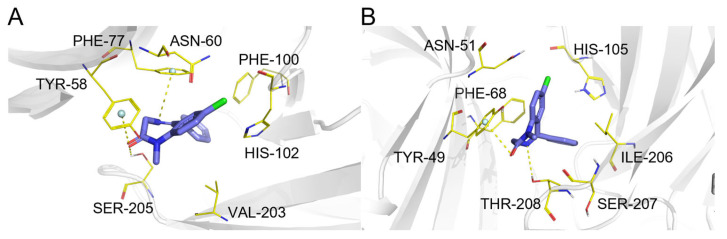
(**A**) Binding mode of diazepam and GABRB2. (**B**) Binding mode of diazepam and GABRA5. Color coding: protein: gray cartoon; key residues: yellow lines; diazepam: slate sticks; p-π interactions: palecyan spheres; intermolecular interactions: yellow dashed lines.

Diazepam (Figure 9, compound **4**) is a benzodiazepine. ComTarget successfully identified and highly ranked its primary therapeutic targets (Table 4), the GABA(A) receptor subunits GABRB2 (9th) and GABRA5 (25th), confirming the tool’s fundamental capability to prioritize established, high-affinity drug targets (Category I). The binding poses for GABRB2 and GABRA5 are shown in Figure 13A and Figure 13B, respectively. 

Notably, ComTarget also predicted interactions with targets exhibiting validated, moderate-affinity binding (Category II). These include bromodomain-containing protein 4 (BRD4), with an experimental IC_50_ of ~7 µM. This prediction aligns with an independent virtual screening study that identified diazepam as a selective inhibitor of BRD4, suggesting a potential epigenetic mechanism for this classic drug beyond its CNS effects [55]. Additionally, CA2 has a Ki of 0.58 µM (Category II). This interaction is not only robust in vitro but has also been shown to induce significant enzymatic inhibition in vivo following intravenous administration at a pharmacologically relevant dose (2 mg/kg) [56].

#### 2.5.5. Atorvastatin

**Figure 14:**
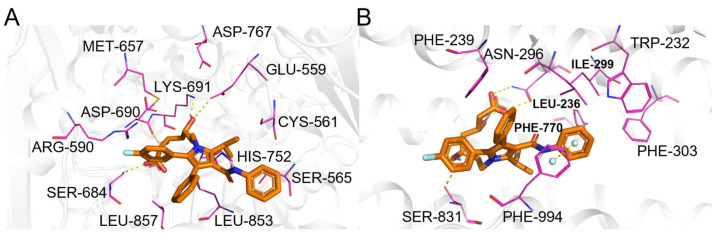
(**A**) Binding mode of atorvastatin and 3-hydroxy-3-methylglutaryl-coenzyme A reductase. (**B**) Binding mode of atorvastatin and ATP-dependent translocase ABCB1. Color coding: protein: gray cartoon; key residues: magenta lines; atorvastatin: orange sticks; π-π interactions: palecyan spheres; intermolecular interactions: yellow dashed lines.

Atorvastatin (Figure 9, compound **5**) is a lipid-lowering drug whose primary target is HMG-CoA reductase. Correctly, ComTarget identified and prioritized this primary therapeutic target (Table 5), binding mode (Figure 14A), which achieved the top rank in the similarity-based sorting (Category I), underscoring the efficacy of the shape-similarity pre-screening step.

The tool also identified validated secondary targets. Notably, the efflux transporter ABCB1 (P-glycoprotein) was ranked 8th (Category II), with a binding mode (Figure 14B). This well-documented interaction is crucial for understanding atorvastatin’s drug–drug interaction potential and resistance mechanisms. Predictions for lower-affinity potential interactions (Category III) included cholinesterase [59], supported by in vivo inhibition at very high doses, highlighting the tool’s ability to generate hypotheses about off-target effects under specific conditions.

#### 2.5.6. Berberine

Berberine (Figure 9, compound **6**) is a natural isoquinoline alkaloid with broad pharmacological activities.

**Figure 15:**
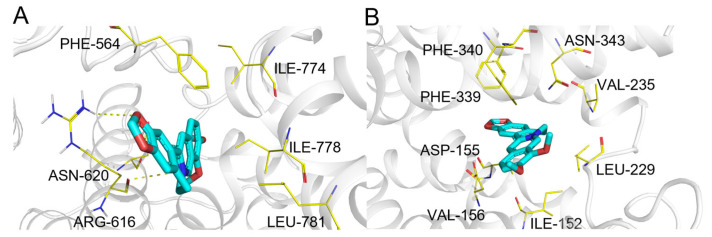
(**A**) Binding mode of berberine and PDE5. (**B**) Binding mode of berberine and 5-hydroxytryptamine receptor 2A. Color coding: protein: gray cartoon; key residues: yellow lines; berberine: cyan sticks; intermolecular interactions: yellow dashed lines.

The predictions encompassed targets with low to moderate direct binding affinity (Table 6, Categories II & III). For example, PDE5 and ABCG2 were top-ranked but are supported by evidence from plant extracts with weak inhibitory activity (Category III). Representative binding modes for PDE5 and the 5-HT2A receptor (HTR2A) are shown in Figure 15A,B, illustrating plausible molecular interactions.

Notably, ComTarget highly ranked several targets for which strong functional pharmacological evidence exists (Category F), despite the absence of published binding constants. These include the adenosine A2A receptor (ADORA2A) and the 5-HT2A receptor (HTR2A), where independent in vivo studies have shown that their blockade abolishes key pharmacological effects of berberine, such as anti-fibrotic and antidepressant-like activities [63,64]. This case illustrates the tool’s utility for pleiotropic compounds, provided computational rankings are integrated with diverse evidence types encompassing biochemical affinity to functional necessity.

#### 2.5.7. Cryptotanshinone

Cryptotanshinone (Figure 9, compound **7**) is a natural product with reported antitumor activities. ComTarget’s predictions for cryptotanshinone yielded targets across different evidence categories, demonstrating its comprehensive screening capability (Table 7).

The estrogen receptor was predicted as a high-confidence target (Category I). This prediction aligns with the compound’s potential role in modulating hormone receptor pathways implicated in tumor progression.

**Figure 16:**
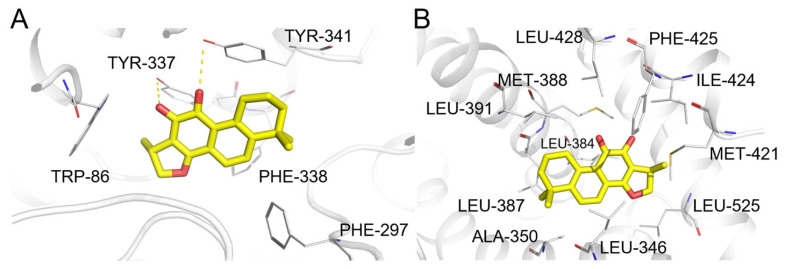
(**A**) Binding mode of cryptotanshinone and acetylcholinesterase. (**B**) Binding mode of cryptotanshinone and androgen receptor. Color coding: protein: gray cartoon; key residues: gray lines; cryptotanshinone: yellow sticks; intermolecular interactions: yellow dashed lines.

Additionally, ComTarget identified Acetylcholinesterase (AChE) (ranked 33rd, Category III). While AChE is primarily associated with neurotransmission, its involvement in tumor cell processes has been reported, suggesting a potential secondary mechanism. The weaker evidence category (III) indicates this interaction may be of lower affinity or requires further validation at pharmacologically relevant concentrations. The predicted binding modes for these targets are visualized in Figure 16A,B.

This case study evaluated ComTarget using seven representative small molecules, spanning classic drugs and natural products. The results demonstrate that ComTarget can provide a multi-tiered target prediction profile. It thus serves as a comprehensive tool that maps a multi-dimensional target landscape from chemical structure, effectively aiding in the exploration of mechanisms of action, especially for multi-target drugs and natural products.

### 2.6. Comparison with the Similarity Ensemble Approach (SEA)

To evaluate the performance of ComTarget against established ligand-based methods, we used the set of fluoxetine targets annotated in the IUPHAR/BPS Guide to PHARMACOLOGY (GtoPdb) as a reference benchmark, and systematically compared the predictions from SEA and ComTarget (Table 8). An extended comparison based on annotations from the ChEMBL database is also provided (Supplementary Table S3). The analysis shows that both methods effectively identify core therapeutic targets, such as the serotonin transporter (SERT) and the 5-HT2A receptor. However, their prediction profiles differ significantly, highlighting a fundamental methodological distinction. SEA successfully predicted several other monoamine transporters (e.g., NET, DAT) and 5-HT receptor subtypes (e.g., 5-HT2C, 5-HT6), owing to its extensive knowledge base of known ligand-target interactions. In contrast, ComTarget did not predict NET and DAT, primarily because high-quality 3D complex structures suitable for reverse docking are lacking for these targets, and thus they were not included in ComTarget’s structure library. ComTarget predicted potential targets not identified by SEA, such as acetylcholinesterase (AChE), the muscarinic M3 receptor, CA2, and bromodomain-containing protein 4 (BRD4) (in the ChEMBL comparison). Among these, AChE and CA2 have been associated with fluoxetine’s metabolism or side effects. This outcome underscores the characteristic of ComTarget as a 3D structure-driven tool: it does not rely on the chemical profiles of known ligands but identifies potential interaction patterns through shape similarity. Consequently, ComTarget enables “scaffold-hopping” discovery by uncovering novel target hints through the recognition of complementary 3D spatial and physicochemical properties, even among compounds with distinct topological scaffolds.

## 3. Discussion

This study presents ComTarget, a computational tool that integrates three-dimensional molecular shape similarity (C3DD) with reverse docking to predict potential protein targets for small molecules. Evaluation on the DEKOIS 2.0 and DUDE-Z benchmark datasets demonstrated that the C3DD method provides a unique performance profile. The C3DD method leverages combined 3D shape descriptors to capture both global and local molecular features without the need for structural superposition. This approach enhances the discrimination of compounds that share similar bioactive conformations but possess distinct chemical scaffolds, highlighting its potential for scaffold-hopping applications. It consistently outperformed conventional 2D fingerprint methods by successfully capturing 3D shape complementarity [76], enabling the identification of active compounds with dissimilar scaffolds, which is a key advantage for scaffold hopping [77]. Our benchmarking on the DEKOIS 2.0 and DUDE-Z datasets demonstrated that C3DD consistently and significantly outperformed conventional 2D fingerprint methods (ECFP4, MACCS) and showed competitive or advantageous performance on specific targets where 3D shape complementarity is crucial (e.g., ACE2, AURKB, KIF11, PUR2). The efficient, alignment-free nature of C3DD enables rapid similarity screening, effectively enriching true actives within the top ranks of a large library. This serves as a powerful and computationally efficient pre-filter, significantly streamlining the subsequent, more resource-intensive reverse docking process.

ComTarget integrates ligand-based (e.g., TargetHunter [8], CSNAP3D [11]) and receptor-based (e.g., idTarget [22], TarFisDock [23]) strategies to address limitations of individual approaches. Unlike web services like SwissTargetPrediction and PharmMapper [12,29], ComTarget is implemented locally in the C programming language, providing enhanced efficiency and flexibility in customizing resources and parameters. This renders it applicable to tasks such as large-scale batch processing, drug repositioning, and adverse reaction prediction. Furthermore, case studies were performed with FDA-approved drugs (e.g., Imatinib, Aspirin) and natural products (e.g., Berberine). ComTarget was able to recapitulate known primary therapeutic targets and identify potential off-targets associated with pharmacological mechanisms and side effects. These results validate its potential utility in polypharmacology research. Compared with ligand-based methods such as SEA, ComTarget exhibits a different prediction method. SEA relies on the chemical similarity of known ligands to infer targets, achieving high recall for well-characterized target families. ComTarget is structure-driven: its predictions depend on the availability of 3D complex structures and shape complementarity between query molecules and binding pockets. This enables the identification of targets without requiring prior ligand annotations, as exemplified by its prediction of acetylcholinesterase and CA2 for fluoxetine, but also means its coverage is constrained by the structural data available in the PDB. This complementarity suggests that ComTarget is capable of uncovering scaffold-hopping opportunities that may be missed by methods relying solely on known chemical space.

In the case studies, we noted the recurrent appearance of CA2 in the prediction lists for multiple drugs. This phenomenon warrants discussion, as it may reveal certain systematic characteristics of the computational method or the underlying database. We analyzed and identified three primary reasons: First, structural database composition bias: CA2 is one of the most extensively and highly resolved proteins in the PDB (with over 600 records). Its abundance of high-quality, ligand-bound complex structures significantly increases its prior probability of being matched during similarity-based searches. Second, physicochemical properties of the binding pocket: CA2 possesses a deep, conserved hydrophobic pocket enriched with polar and metal-ion features [78]. This characteristic may confer non-specific computational affinity for many drug-like molecules containing aromatic rings and polar groups [79]. Third, sensitivity of the shape descriptors: The 3D shape descriptors employed in our method may be particularly sensitive to such regular, compact, cave-like structures. Therefore, the frequent appearance of CA2 should be interpreted primarily as a reflection of these methodological and database attributes, and such results warrant cautious interpretation and prioritization for experimental validation.

ComTarget has several limitations despite its potential applications. The reverse docking module is computationally intensive, and processing large-scale target libraries is computationally time-consuming. This is a common challenge for molecular docking-based methods. The target library relies solely on experimental structures from the PDB, which may omit important targets that lack experimentally determined structural data (e.g., certain membrane proteins or understudied targets). Furthermore, prediction outputs are sensitive to the quality of input 3D molecular conformations; errors may arise when input 3D molecular conformations are not adequately optimized or if molecular conformation sampling is inadequate. For future work, we aim to accelerate reverse docking through parallel computing or machine learning approaches. We also plan to integrate predicted protein structures from AlphaFold3 to broaden target coverage, which could improve the tool’s performance and applicability [80]. ComTarget is currently distributed as a command-line program; its core makes it readily adaptable for integration into web-based platforms or local graphical user interfaces. We plan to develop such an interface in future versions to enhance accessibility.

In summary, ComTarget provides a practical local solution for small-molecule target prediction by integrating 3D molecular shape similarity and reverse docking. It effectively identifies therapeutic targets and off-targets, showing potential for applications in polypharmacology, drug repositioning, and adverse effect prediction. As a free, open-source tool, ComTarget has the potential to act as a useful resource for deciphering complex drug action mechanisms and aiding in the discovery of multi-target drugs.

## 4. Materials and Methods

### 4.1. File Input

The ComTarget program accepts 3D molecular structures in MOL2, PDB, SDF, and XYZ formats. To minimize calculation errors, input 3D structures should be optimized before use, with the primary goal of eliminating unreasonable bond lengths and bond angles. For most small molecules, molecular mechanics force fields are sufficiently accurate and computationally efficient. Recommended approaches include Open Babel (version 3.0.0 or later) [81] with the MMFF94 force field using 5000 minimization steps, or Avogadro (version 2.0.0) [82] (a free graphical molecular editor) with GAFF, MMFF94, MMFF94s, or UFF force fields. However, for molecules where force-field optimization fails to produce reasonable geometries, semi-empirical or quantum-chemical methods are recommended. These include semi-empirical calculations using MOPAC2016 [83] (PM6-D3H4 [84] OPT), or density functional theory (DFT) using ORCA (version 4.0 or later) [85] at the r2SCAN-3c [86,87,88] functional/basis set. In practice, force field optimization is sufficient for the vast majority of routine applications to achieve reliable shape similarity and docking results. Only when force field minimization yields clearly unrealistic conformations (e.g., distorted rings or highly non-planar conjugated systems) should users resort to semi-empirical or DFT methods. Users are encouraged to choose the method that best balances accuracy and computational cost for their specific query molecule.

### 4.2. Conformational Search

For input 3D structures, conformational search is context-dependent. For the reference ligand database (~2600 targets), each ligand’s original conformation was taken directly from its PDB structure without further modification, preserving the experimental bioactive pose.

For a user-supplied query molecule, conformational flexibility is handled through a guided workflow. The number of rotatable bonds is first computed; if ≥5, skipping conformer generation is recommended to avoid combinatorial explosion. For more rigid queries (<5 rotatable bonds), conformers are generated via Open Babel (version 3.0.0 or later) [81] using either the Monte Carlo method (up to 500 conformers) or the Confab algorithm (with an energy cutoff of 50.0 kcal/mol and iterative RMSD reduction) [89]. All generated conformers are energy-minimized with the MMFF94 force field (3000 steps). A Boltzmann distribution at 298.15 K is then calculated, and conformers above a user-defined population threshold (default 0.5%) are retained. This typically yields 1–30 conformers per query, capped at 100 for downstream efficiency.

Thus, the database contains static experimental conformations, while queries are represented by an ensemble of relevant low-energy conformations.

### 4.3. Target Library Preparation

A library of protein-ligand complex structures is curated from the Protein Data Bank (PDB) [90]. Following data cleaning, a final set of 26,272 complexes was retained, corresponding to 4429 unique protein targets (where multiple PDB structures may represent a single protein). The size of the ComTarget library (4429 targets) is comparable to or larger than that of widely used tools (e.g., SwissTargetPrediction, which covers >2000 targets); a comprehensive comparison is provided in Table S1. To characterize the functional composition of the curated library, protein targets were categorized based on their primary biological functions. As summarized in Table S2, enzymes constitute the largest category (33.78%), followed by signaling proteins (17.70%). This functional diversity ensures broad coverage for subsequent reverse docking and similarity analysis.

### 4.4. Molecular Similarity Descriptor Calculation

A set of ten 3D shape descriptors, including Hu moment invariants and compactness-related measures, was selected. These descriptors provide complementary characterizations of molecular shape from both global and local perspectives. For instance, Hu moments are invariant to rotation, translation, and scaling, offering a robust description of the overall shape, while descriptors like compactness quantify specific local geometric properties. This combination addresses a key limitation of conventional 2D fingerprint-based methods, which rely primarily on substructure similarity and may fail to identify compounds that share similar 3D shapes despite having distinct chemical scaffolds. Preliminary experiments and comparative results (see the Results section) confirm that this descriptor set outperforms traditional 2D fingerprints in shape similarity tasks, validating our selection.

For the input 3D molecular structures, molecular grid data were first generated. Based on these grid data, molecular shape descriptors were computed, including molecular volume and surface area, as described below.

Geometric moments have been widely applied in 2D and 3D image processing and computer vision [91,92]. As described in reference for a given 3D shape *S*, (*p,q,r*)-moment denoted as *m_p,q,r_*(*S*) is defined as Equation (1) [93].

(1) $$m_{p,q,r}(S)=\int \int \int x^{p}y^{q}z^{r}dxdydz$$

*μ_p,q,r_*(*S*) is called the centralized (*p,q,r*)-moment of 3D shape *S* as shown in Equation (2).

(2) $$\mu_{p,q,r}(S)=\int \int \int (x-\bar{x}(S){)}^{p}{\left(y-\bar{y}(S)\right)}^{q}{\left(z-\bar{z}(S)\right)}^{r}dxdydz$$

The order of *m_p,q,r_*(*S*) and *μ_p,q,r_*(*S*) is *p* + *q* + *r*. And the centroid $\left(\bar{x}(S),\bar{y}(S),\bar{z}(S)\right)$ is calculated as Equation (3) [93].

(3) $$(\bar{x}(S),\bar{y}(S),\bar{z}(S))=\left(\frac{m_{1,0,0}(S)}{m_{0,0,0}(S)},\frac{m_{0,1,0}(S)}{m_{0,0,0}(S)},\frac{m_{0,0,1}(S)}{m_{0,0,0}(S)}\right)$$

Four 3D Hu moment invariants *τ*_1_(*S*), *τ*_2_(*S*), *τ*_3_(*S*), *τ*_4_(*S*) are defined as Equations (4)–(7) [91,92].

(4) $$\tau_{1}(S)=\mu_{2,0,0}(S)+\mu_{0,2,0}(S)+\mu_{0,0,2}(S)$$

(5) $$\tau_{2}(S)=\mu_{2,0,0}(S)\cdot \mu_{0,2,0}(S)+\mu_{2,0,0}(S)\cdot \mu_{0,0,2}(S)+\mu_{0,2,0}(S)\cdot \mu_{0,0,2}(S)-{\mu^{2}}_{1,1,0}(S)-{\mu^{2}}_{1,0,1}(S)-{\mu^{2}}_{0,0,1}(S)$$

(6) $$\begin{array}{l} \tau_{3}(S)=\mu_{2,0,0}(S)\cdot \mu_{0,2,0}(S)\cdot \mu_{0,0,2}(S)+2\cdot \mu_{1,1,0}(S)\cdot \mu_{1,0,1}(S)\cdot \mu_{0,1,1}(S)-\mu_{2,0,0}(S)\cdot {\mu^{2}}_{0,1,1}(S)-\mu_{0,2,0}(S)\cdot {\mu^{2}}_{1,0,1}(S)\\ -\mu_{0,0,2}(S)\cdot {\mu^{2}}_{1,1,0}(S) \end{array}$$

(7) $$\begin{array}{l} \tau_{4}(S)=\mu_{3,0,0}^{2}(S)+\mu_{0,3,0}^{2}(S)+\mu_{0,0,3}^{2}(S)+3\cdot \mu_{1,2,0}^{2}(S)+3\cdot \mu_{1,0,2}^{2}(S)+3\cdot \mu_{0,1,2}^{2}(S)+3\cdot \mu_{2,1,0}^{2}(S)+3\cdot \mu_{0,2,1}^{2}(S)+3\cdot \mu_{2,0,1}^{2}(S)\\ +6\cdot \mu_{1,1,1}^{2}(S) \end{array}$$

3D shape compactness measure *κ*(*S*), *κ*_st_(*S*), and the compactness measure *κ*_fit_(*S*) are defined as Equations (8)–(10) [93].

(8) $$\kappa (S)=\frac{3^{5/3}}{5{\left(4\pi \right)}^{2/3}}\cdot \frac{1}{\tau_{1}(S)}$$

(9) $$\kappa_{\mathrm{st}}(S)=\frac{36\cdot \pi \cdot {\left(volume\_of\_S\right)}^{2}}{{\left(surface\_area\_of\_S\right)}^{3}}$$

(10) $$\kappa_{\mathrm{fit}}(S)=\frac{volume(S\cap FS(S))}{volume(S\cup FS(S))}$$

The surface area (*S*) to volume (*V*) ratio (denoted as *R*) is defined as Equation (11).

(11) $$R=\frac{S}{V}$$

In this study, a total of 10 combined 3D molecular shape descriptors (C3DD) is used for 3D molecular similarity calculation: *τ*_1_(*S*), *τ*_2_(*S*), *τ*_3_(*S*), *τ*_4_(*S*), *κ*(*S*), *κ*_st_(*S*), *κ*_fit_(*S*), *S*, *V*, *R*. Among these, the *S*, *V*, and the point cloud data representing the molecular shape were calculated using the relevant modules of CalVSP, a previously published computational tool [94]. Several core descriptors in C3DD (*κ*(*S*), *κ*_st_(*S*), *κ*_fit_(*S*), *S*, *V*, *R*) are directly derived from molecular surface areas, volumes, and compactness measures. Consequently, molecules deemed “similar” by C3DD inherently share comparable 3D size and overall shape profiles.

### 4.5. 3D Molecular Similarity Comparison

The degree of molecular shape similarity is measured by calculating the differences between molecular shape descriptors. Moments, as molecular shape descriptors, possess rotation-independent properties. Therefore, the similarity between any two molecules A (*A* = [*α*_1_, α_2_, …, *α*_10_]) and B (*B* = [*β*_1_, *β*_2_, …, *β*_10_]) can be computed without superposition operations. The similarity score is defined as Equation (12).

(12) $$Similarity\left(A,B\right)=\sum_{i=1}^{i=10}\frac{\min(\alpha_{i},\beta_{i})}{\max(\alpha_{i},\beta_{i})}\cdot \frac{1}{10}.\;(\max(\alpha_{i},\beta_{i})\neq 0)$$

**Figure 17:**
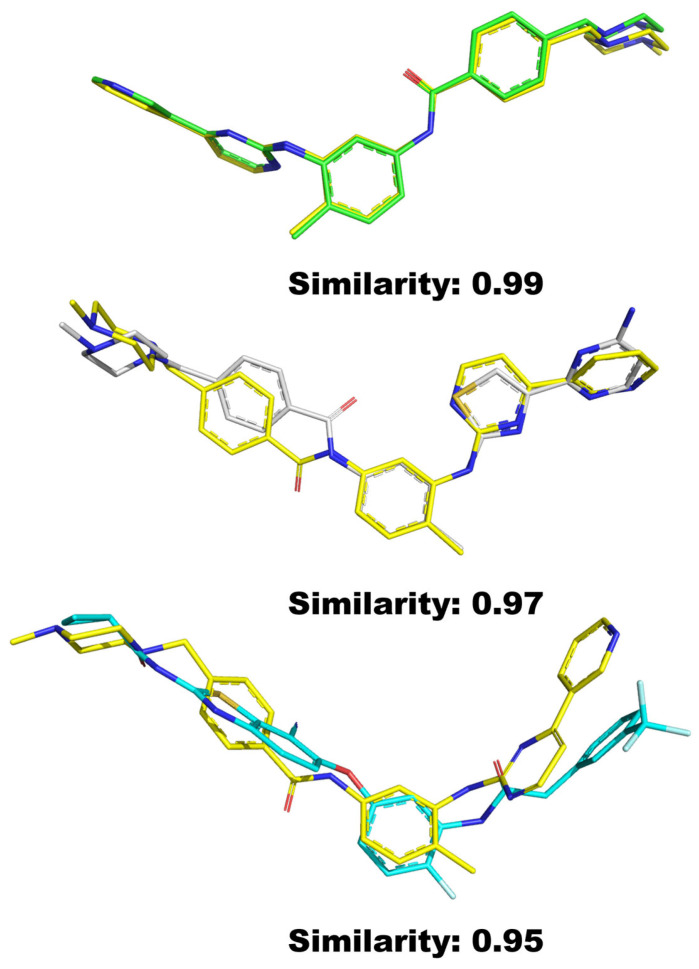
Compounds similar to the 3D structure of imatinib (yellow) were identified through the C3DD molecular similarity calculation method. Color coding: imatinib: yellow; other compounds: green, gray, and cyan (colors are used only for visual distinction among different comparison molecules).

We calculate the similarity between the query small molecules (which require target identification) and the small molecules in reported protein-ligand complexes. As Figure 17 shows, the compounds are sorted based on their similarity scores to identify those that are structurally similar at the 3D conformational level, thereby enabling the determination of potential binding targets. Finally, reverse docking calculations are performed on the ranked targets.

### 4.6. Reverse Docking Calculation

First, the collected PDB protein structure files are processed using the prepare_receptor4.py script from the mgltools (version 1.5.7); however, because some Python (version 3.8.8) scripts in this version may produce errors, we manually process the structures using the AutoDockTools package (version 1.5.6 Sep_17_14) [95] when needed. This step involves adding polar hydrogens and assigning Gasteiger charges, followed by converting the files into the PDBQT format suitable for reverse docking. The small-molecule ligands extracted from the reported PDB structures are used to define the docking site. The docking box dimensions are set according to established protocols described in reference [96]. Subsequently, AutoDock Vina (version 1.1.2 (11 May 2011)) is employed to compute the docking scores for the query small molecules [97].

### 4.7. Reverse Docking Result Sorting

Direct comparison of raw docking scores is inadequate for ranking compounds across different protein targets, primarily due to significant variations in score ranges across different protein-ligand systems. For instance, when using AutoDock Vina to dock a native ligand to its protein target, the resulting scores typically range from −42.2 to −1.5 kcal/mol, depending on the specific system. To correct for systematic variations and enable meaningful cross-target comparison, we adopted a normalized scoring approach. Specifically, for each protein target, the docking score of the query compound (*ScoreA*) is normalized against the docking score of its native ligand (*ScoreB*). The normalized differential score (*DetScore*) is calculated as follows (Equation (13)).

(13) DetScore=ScoreA−ScoreB

A smaller (i.e., more negative) *DetScore* indicates that, relative to the native ligand, the query compound achieved a more favorable docking score, which suggests a potentially higher predicted binding affinity. Consequently, a negative *DetScore* implies that the query compound is predicted to bind more strongly to the target than the native ligand does.

All reverse docking results are then sorted in ascending order of *DetScore* (from most negative to least negative/positive). This ranking approach corrects for system-specific score variations and directly prioritizes targets for which the query compound shows the greatest relative binding improvement, thereby enabling efficient and unbiased screening of potential protein targets.

## 5. Conclusions

This study introduces ComTarget, a computational tool that integrates 3D molecular similarity search with molecular docking to predict potential molecular targets. This integrated approach holds significant value for advancing polypharmacology studies and adverse effect prediction.

In the similarity search component, the C3DD method, based on combined 3D shape descriptors, demonstrated its capability to effectively capture 3D molecular shape complementarity. Benchmarking on the DEKOIS 2.0 and DUDE-Z datasets confirmed that C3DD outperforms conventional 2D fingerprint methods and serves as an efficient computational filter to enrich candidate targets before reverse docking. Evaluation on diverse test compounds, including FDA-approved drugs and natural products, confirmed that ComTarget reliably identifies both therapeutic targets and those associated with adverse effects, underscoring its practical utility in multi-target profiling.

However, the molecular docking module of ComTarget is limited by computational intensity, leading to prolonged processing times. Improving the efficiency of this component will be a key focus for future work.

## Figures and Tables

**Table 1:** The protein target candidates of Imatinib identified by ComTarget.

Rank *^a^	PDB ID	Evidence Category ^†^	Targets
1/51	1IEP	II	ABL1 [37]
7/3	4BKJ	II	DDR1 [38]
10/13	6JOL	I	PDGFRA [39]
11/146	3F3V	II	SRC [40]
12/9	4KSP	II	BRAF *^b^
13/19	2PL0	I	LCK [41]
26/193	8A2B	II	EGFR *^c^
30/177	3BV3	III	MAPK14 [42]
31/169	5K00	II	MELK *^d^
58/7	1T46	II	KIT [40]
75/93	6VXH	II	ABCG2 *^f^
97/24	4WHZ	II	MAPK10 [43]
103/200	7UY0	II	FGR *^g^
152/174	8X5M	II	MAPK8 *^h^
158/41	7QRK	I	CA2 *^i^

*a, Rank: comprehensive sorting (first number)/similarity sorting (second number). ^†^ Evidence Category: I (High-affinity target): Kd/IC_50_/EC_50_ ≤ 100 nM. II (Moderate-affinity target): 100 nM < Kd/IC_50_/EC_50_ ≤ 10,000 nM. III (Low-affinity target): Kd/IC_50_/EC_50_ > 10,000 nM or preliminary evidence. *b, BindingDB Entry https://doi.org/10.7270/Q25D8S70. *c, BindingDB Entry https://doi.org/10.7270/Q29W0CTP. *d, BindingDB Entry https://doi.org/10.7270/Q2TM78GH. *f, BindingDB Entry https://doi.org/10.1038/s41467-020-16155-2. *g, BindingDB Entry https://doi.org/10.7270/Q2JD4V4C. *h, BindingDB Entry https://doi.org/10.7270/Q2BK19Q3. *i, BindingDB Entry https://doi.org/10.1016/j.bmcl.2009.06.002.

**Table 2:** The protein target candidates of Aspirin identified by ComTarget.

Rank *^a^	PDB ID	Evidence Category ^†^	Targets
6/135	7Y2A	III	CA2 [44]
21/85	6NTO	III	ACHE [45]
54/58	7U8H	III	KRAS *^b^
60/43	1OXR	II	PLA2 [46]
140/86	8J3W	III	ABCC4 *^c^ [47]

*a, Rank: comprehensive sorting (first number)/similarity sorting (second number). ^†^ Evidence Category: I (High-affinity target): Kd/IC_50_/EC_50_ ≤ 100 nM. II (Moderate-affinity target): 100 nM < Kd/IC_50_/EC_50_ ≤ 10,000 nM. III (Low-affinity target): Kd/IC_50_/EC_50_ > 10,000 nM or preliminary evidence. *b, BindingDB Entry https://doi.org/10.7270/Q2D21W1M. *c, BindingDB Entry https://doi.org/10.7270/Q2JM2D2D.

**Table 3:** The protein target candidates of Fluoxetine identified by ComTarget.

Rank *^a^	PDB ID	Evidence Category ^†^	Targets
11/140	8X63	II	HRH1 [48]
18/14	6VRH	I	SLC6A4 [49]
23/134	4MM4 *^b^	II	bacterial leucine transporter (LeuBAT) [50]
98/137	6OUJ	II Activation	CA2 *^c^ [51]
74/3	7WKZ	III	ALB [52]

*a, Rank: comprehensive sorting (first number)/similarity sorting (second number). ^†^ Evidence Category: I (High-affinity target): Kd/IC_50_/EC_50_ ≤ 100 nM. II (Moderate-affinity target): 100 nM < Kd/IC_50_/EC_50_ ≤ 10,000 nM. III (Low-affinity target): Kd/IC_50_/EC_50_ > 10,000 nM or preliminary evidence. *b, PDB 4MM4 is an engineered bacterial leucine transporter (LeuBAT) designed to mimic the pharmacology of human biogenic amine transporters. The prediction indicates recognition of the conserved binding mode of fluoxetine within this transporter family. *c, Fluoxetine acts as a potent activator (not inhibitor) of CA2 at clinically relevant concentrations (~1 µM).

**Table 4:** The protein target candidates of Diazepam identified by ComTarget.

Rank *^a^	PDB ID	Evidence Category ^†^	Targets
9/45	6X3X	III	GABRB2 [53]
25/24	8BHK	II	GABRA5 [54]
52/157	6UWX	II	BRD4 [55]
81/170	1EOU	II	CA2 [56]

*a, Rank: comprehensive sorting (first number)/similarity sorting (second number). ^†^ Evidence Category: I (High-affinity target): Kd/IC_50_/EC_50_ ≤ 100 nM. II (Moderate-affinity target): 100 nM < Kd/IC_50_/EC_50_ ≤ 10,000 nM. III (Low-affinity target): Kd/IC_50_/EC_50_ > 10,000 nM or preliminary evidence.

**Table 5:** The protein target candidates of Atorvastatin identified by ComTarget.

Rank *^a^	PDB ID	Evidence Category ^†^	Targets
8/156	8Y6I	II	ABCB1 [57]
11/47	6U7P	III	Protease [58]
15/105	6I0B	III	Cholinesterase [59]
22/1	2Q1L	I	HMGCR [60]

*a, Rank: comprehensive sorting (first number)/similarity sorting (second number). ^†^ Evidence Category: I (High-affinity target): Kd/IC_50_/EC_50_ ≤ 100 nM. II (Moderate-affinity target): 100 nM < Kd/IC_50_/EC_50_ ≤ 10,000 nM. III (Low-affinity target): Kd/IC_50_/EC_50_ > 10,000 nM or preliminary evidence.

**Table 6:** The protein target candidates of Berberine identified by ComTarget.

Rank *^a^	PDB ID	Evidence Category ^†^	Targets
1/153	6VBI	III	PDE5 [61]
2/67	7OJ8	III	ABCG2 [62]
3/16	6ZDV	F	ADORA2A [63]
8/126	6WGT	F	HTR2A [64]
10/172	7BK1	F	CHEK1 [65]
12/17	3BTI	II	QacR [66]
29/68	3E64	F	JAK2 [67]
36/178	7C7H	III	AKR1C3 [68]
83/49	4N8E	III	DPP4 [69]
82/32	6U9V	III	P2RX7 [70]
92/188	3SVV	F	SRC [71]
110/45	2XGS	II	OGT [72]
159/168	2XDW	III	PREP [73]

*a, Rank: comprehensive sorting (first number)/similarity sorting (second number). ^†^ Evidence Category: I (High-affinity target): Kd/IC_50_/EC_50_ ≤ 100 nM. II (Moderate-affinity target): 100 nM < Kd/IC_50_/EC_50_ ≤ 10,000 nM. III (Low-affinity target): Kd/IC_50_/EC_50_ > 10,000 nM or preliminary evidence. F Functional evidence without reported binding affinity.

**Table 7:** The protein target candidates of Cryptotanshinone identified by ComTarget.

Rank *^a^	PDB ID	Evidence Category ^†^	Targets
33/117	4B82	III	ACHE [74]
92/58	4PXM	I	ESR1 [75]

*a, Rank: comprehensive sorting (first number)/similarity sorting (second number). ^†^ Evidence Category: I (High-affinity target): Kd/IC_50_/EC_50_ ≤ 100 nM. II (Moderate-affinity target): 100 nM < Kd/IC_50_/EC_50_ ≤ 10,000 nM. III (Low-affinity target): Kd/IC_50_/EC_50_ > 10,000 nM or preliminary evidence.

**Table 8:** Comparison of the Similarity Ensemble Approach (SEA) and ComTarget.

Targets *^a^	SEA *^b^	Comtarget
HTR2A	True *^c^	True
HTR2C	True	
HTR6	True	
ACHE		True
ADRA2A		
ADRA2B		
CYP2D6	True	
DRD2		
HRH3		
CHRM1		
CHRM3		True
CHRM5		
SLC6A2		
KCNK2		
TMEM97		
SIGMAR1		
SLC6A3	True	
SLC6A2	True	
SLC6A4	True	True
Transporter		
CACNA1C		
KCNH2		
KCNC1		
HTR2B		
KCNJ6		
SLC29A4		

*a, Comparison based on annotations from the IUPHAR/BPS Guide to PHARMACOLOGY (GtoPdb) database. *b, data obtained from https://sea.bkslab.org/ *c, True represents correctly identifying the target.

## Data Availability

DEKOIS 2.0 dataset is obtained from http://www.dekois.com (accessed on 25 April 2026). The DUDE-Z dataset is obtained from https://dudez.docking.org/ (accessed on 25 April 2026). Shapescreen is obtained from https://cdpkit.org/applications/index.html (accessed on 25 April 2026). ROSHAMBO is obtained from github.com/molecularinformatics/roshambo (accessed on 25 April 2026). The software tools used in this study, including Open Babel 3.0.0–7 October 2019--20:03:12 (https://github.com/openbabel/openbabel/releases), Autodock Vina 1.1.2. win32 (https://autodock-vina.readthedocs.io/ (accessed on 25 April 2026)), Python IDE Spyder (https://www.spyder-ide.org/ (accessed on 25 April 2026)). ComTarget source code and usage are freely available from https://github.com/CalVSP/ComTarget.git (accessed on 25 April 2026). Receptor data of ComTarget is available from https://figshare.com/articles/dataset/Reverse_Target_Fishing_Protein_PDBQT_Database/30812579 (accessed on 25 April 2026). Further inquiries can be directed to the corresponding author.

## Supplementary Materials

The following supporting information can be downloaded at: https://www.mdpi.com/article/10.3390/ph19050715/s1, Table S1 (comparison of target library sizes across computational tools); Table S2 (functional classification of protein targets in the ComTarget library); Table S3 (Comparison of Similarity Ensemble Approach (SEA) and ComTarget). Structure of test cases (ZIP).
