# Supplementary material for: ComTarget: Small-Molecule Target Prediction with Combinatorial Modeling
Source: Pharmaceuticals (Basel). 2026 Apr 30;19(5):715. doi: 10.3390/ph19050715 (PMC13209433; doi:10.3390/ph19050715)
Supplement: Supplementary file 1 [file pharmaceuticals-19-00715-s001.zip › support_information.pdf]

ComTarget: Small-Molecule Target Prediction with Combinatorial Modeling

Yuzhu Li[1], Qingyi Shi[2], Xingjie Lu[2], Daiju Yang[2], Dilixiati Yeerken[2], Huizi Jin[1]*, Qingyan Sun[2]*

1 School of Pharmaceutical Sciences, Shanghai Jiao Tong University, Shanghai 200240, China

2 National Key Laboratory of Lead Druggability Research, Shanghai Institute of Pharmaceutical Industry, China State Institute of Pharmaceutical Industry, China State Institute of Pharmaceutical Industry Co., Ltd., Shanghai 201203, China

* Authors to whom correspondence should be addressed. Correspondence to Qingyan Sun Email: sqy_2000@163.com or Huizi Jin Email: kimhz@sjtu.edu.cn

Table S1. Target Library Sizes of Computational Tools

Name	Targets
SuperPred	1800 targets
TarPred	533 targets
Swiss TargetPrediction	>2000 targets
ChemProt	>20,000 proteins
PharmMapper	23,236 proteins
TarFisDock	1207 targets
idTarget	All protein structures in the PDB
INVDOCK	9000 entries
ComTarget	4429 targets 23,998 protrins 26,272 ligands

Table S2. Functional Classification of Protein Targets in the Library

Category	Counts	Total Percentage
Enzyme	1496	33.78%
Signaling Protein	784	17.70%
Antibody/Immunoglobulin	348	7.86%
Transporter	314	7.09%
Transcription Factor	285	6.43%
Receptor	253	5.71%
Structural Protein	231	5.22%
Ion Channel	200	4.52%
Other Functional Proteins	273	6.16%
Unclassified	245	5.53%

Table S3. Comparison of Similarity Ensemble Approach (SEA) and ComTarget[*a]

SEA[*b]	ComTarget
5-hydroxytryptamine receptor 2A	5-hydroxytryptamine receptor 2A
Sodium-dependent serotonin transporter	Sodium-dependent serotonin transporter
5-hydroxytryptamine receptor 2C	3',5'-cyclic-AMP phosphodiesterase 4D
5-hydroxytryptamine receptor 2C	5-hydroxytryptamine receptor 5A
5-hydroxytryptamine receptor 6	Acetylcholinesterase
Beta-2 adrenergic receptor	Adenosine receptor A2a
Cytochrome P450 2C19	Bromodomain-containing protein 4
Cytochrome P450 2D6	Carbonic anhydrase 2

Cytochrome P450 3A4	Casein kinase I isoform delta
Glucagon receptor	cGMP-specific 3',5'-cyclic phosphodiesterase
Nitric oxide synthase	Cyclin-dependent kinase 2
Potassium channel subfamily K member 2	Estrogen receptor
Sodium-dependent dopamine transporter	Histamine H1 receptor
Sodium-dependent noradrenaline transporter	Histone deacetylase 6
Substance-P receptor	Muscarinic acetylcholine receptor M3
Transporter	Muscarinic acetylcholine receptor M4
Voltage-dependent N-type calcium channel subunit alpha-1B	Peptidyl-prolyl cis-trans isomerase NIMA-interacting 1
	Tyrosine-protein kinase Lck

*a, Comparison based on annotations from the ChEMBL database. *b, data obtained from https://sea.bkslab.org/
